# Atrial Fibrillation and Colonic Neoplasia in African Americans

**DOI:** 10.1371/journal.pone.0135609

**Published:** 2015-08-28

**Authors:** Mehdi Nouraie, Vandana Kansal, Cassius Belfonte, Mohammad Ghazvini, Tahmineh Haidari, Anahita Shahnazi, Hassan Brim, Elsayed Z. Soliman, Hassan Ashktorab

**Affiliations:** 1 Cancer Research Center, and Department of Medicine, Howard University College of Medicine, Washington, District of Columbia, United States of America; 2 Department of Medicine, Division of Cardiology, Howard University College of Medicine, Washington, District of Columbia, United States of America; 3 Cancer Research Center, and Department of Pathology, Howard University College of Medicine, Washington, District of Columbia, United States of America; 4 Epidemiological Cardiology Research Center, Department of Epidemiology and Prevention, Wake Forest School of Medicine, Winston Salem, North Carolina, United States of America; 5 Department of Internal Medicine-Cardiology, Wake Forest School of Medicine, Winston Salem, North Carolina, United States of America; University of Pecs Medical School, HUNGARY

## Abstract

**Background:**

Colorectal cancer (CRC) and atrial fibrillation/flutter (AF) share several risk factors including increasing age and obesity. However, the association between CRC and AF has not been thoroughly examined, especially in African Americans. In this study we aimed to assess the prevalence of AF and its risk factors in colorectal neoplasia in an African American.

**Methods:**

We reviewed records of 527 African American patients diagnosed with CRC and 1008 patients diagnosed with benign colonic lesions at Howard University Hospital from January 2000 to December 2012. A control group of 731 hospitalized patients without any cancer or colonic lesion were randomly selected from the same time and age range, excluding patients who had diagnosis of both CRC and/or adenoma. The presence or absence of AF was based upon ICD-9 code documentation. The prevalence of AF in these three groups was compared by multivariate logistic regression.

**Results:**

The prevalence of AF was highest among CRC patients (10%) followed by adenoma patients (7.2%) then the control group (5.4%, P for trend = 0.002). In the three groups of participants, older age (P<0.008) and heart failure (P<0.001) were significantly associated with higher risk of AF. After adjusting for these risk factors, CRC (OR: 1.4(95%CI):0.9–2.2, P = 0.2) and adenoma (OR: 1.1(95%CI):0.7–1.6, P = 0.7) were not significantly associated AF compared to control group.

**Conclusions:**

AF is highly prevalent among CRC patients; 1 in 10 patients had AF in our study. The predictors of AF in CRC was similar to that in adenoma and other patients after adjustment for potential confounders suggesting that the increased AF risk in CRC is explained by higher prevalence of AF risk factors.

## Background

Atrial Fibrillation (AF) is the most common sustained cardiac arrhythmia in clinical practice [1, 2]. Several studies have shown that the prevalence and incidence of AF is less frequent in African Americans compared to their counterpart whites [3–10]. Paradoxically, several risk factors predicting the development of AF including hypertension (HTN), obesity, diabetes mellitus (DM), and congestive heart failure (CHF) are more prevalent in African Americans [1, 11]. This paradox of AF in African Americans is currently unexplained, but more investigations in AF risk factors may shed light on potential explanation [12].

In the past, AF was considered mainly a consequence of cardiac diseases, recently epidemiologic observations and clinical studies show that different cancer including colorectal cancer (CRC), lung and esophageal cancer could also be associated with higher risk for AF [13] and AF was associated with lower survival rate in cancer survivor [14, 15]. CRC is one of the most common forms of human cancers worldwide with approximately 1 million new cases detected every year [16, 17], with a 20% higher incidence rate and 45% higher mortality in African Americans when compared with Caucasian Americans [18]. Several CRC risk factors including age, sex, low socioeconomic status, are shared by AF [18, 19]. Cancer treatment, paraneoplastic manifestations, shared pathogenesis pathways including inflammation could explain the association between CRC and AF [13]. In a case-control study, patients with a CRC diagnosis had an approximately 3-fold higher risk of AF compared with patients admitted for non-neoplastic diseases [20]. In another study in patients admitted for surgery from the same group, prevalence of AF was twice in patients admitted with a diagnosis of CRC compared with those admitted for non-neoplastic surgery [21]. Furthermore, the prevalence of CRC in a large cohort of patients with and without AF was 0.59% and 0.05%, respectively [22].

The lower risk of AF in African American [11] and higher incidence and mortality of CRC in this group justifies study of association between AF and CRC in African American. Nevertheless African American are under-represented in multiracial studies of AF and CRC and population based studies about association between colorectal lesion and AF in African American urban area are sparse. In this study, we sought to assess the prevalence of AF in African American with colonic lesions (including CRC and adenoma) and to examine the risk factors for AF in patients with colonic lesions compared to African American without colonic lesions or other cancer.

## Methods

### Patients

Study was approved by IRB at Howard University. In this study, we reviewed the available patients’ medical chart which didn’t require to take the informed consent. All patients’ record were anonymized and de-identified prior to analysis. All inpatients 40 years and older from January 2000 to December 2012 were identified using medical data from Howard University Hospital, an inner city tertiary institution in Washington DC. The institutional review board of Howard University Hospital approved the study. We identified 527 African American patients with CRC excluding adenoma and 1008 patients who had only adenoma of the colon and rectum excluding CRC. All diagnoses were documented from discharge records during that period. As a control group, 731 non-cancer inpatients without colonic lesions (including CRC or adenoma) or any other cancer were selected randomly from the same study time and patients’ age range. The absence of CRC or colonic lesion were confirmed by patients’ medical record. The presence or absence of atrial fibrillation and flutter cases in these groups were retrieved based upon the International Classification of Disease (ICD-9) codes documentation (AF ICD-9 codes: 427.31&427.32). We included cases with a diagnosis of atrial fibrillation and flutter listed anywhere in the hospital discharge records. Data on potential confounding factors—that directly or indirectly could be associated with an increased risk of atrial fibrillation and flutter [22]- such as hypertension, heart failure, diabetes mellitus, use of alcohol and tobacco- were also retrieved for each encounter.

### Statistical Analysis

Frequency of AF and its potential risk factors were compared between three groups (CRC, adenoma and controls) using Chi-square (for categorical variables) or Analysis of Variance (for age and number of hospital admissions). To assess the risk factors of AF In each group (CRC, adenoma and controls), we compared the demographic and potential clinical risk factors of AF between two groups of patients (AF+ and AF-) to assess the risk factor of AF. We applied logistic regression models to assess the find the independent risk factors of AF in each group. In each model, we entered the variables with P≤0.2 from univariate analysis and we developed a final model by a backward stepwise approach. Then we assessed the overall effect of adenoma and CRC on AF after adjusting for significant predictors of AF in previous analysis in a unified analysis. This study had a Power = 0.5 to detect an adjusted Odds Ratio (OR) = 1.4 for association between CRC and AF. All Analyses were performed in STATA 12.0 (StataCorp., College Station, TX).

## Results

A group of 2266 patients were included in this study. These were distributed in three groups: patients with CRC (n = 527), patients with colon adenomas (n = 1008) and a control population (n = 731). The frequency of AF was determined in all three groups and was higher in older age patients in all three groups.

**Fig 1:**
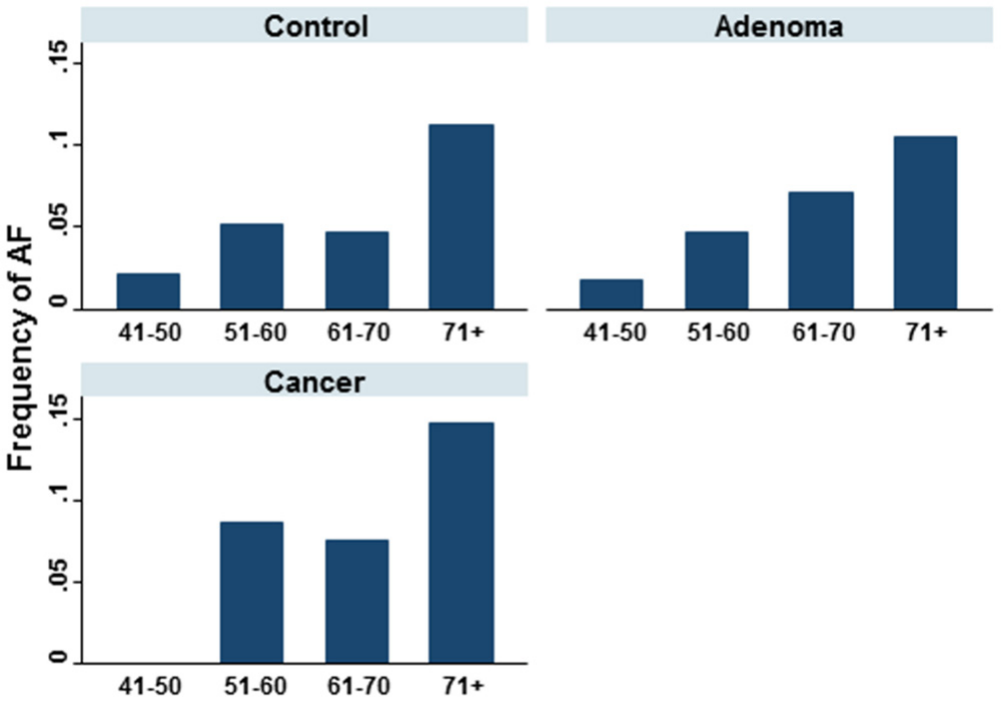
Frequency of AF by age in different groups of patients (P ≤ 0.006).

Table 1 shows the distribution of demographic and clinical variables of CRC, adenoma and control group patients. CRC patients were older compared to controls (median of 67 vs. 56 years in controls, P<0.001). The same applies to adenomas patients when compared to the control group (median 66 vs. 56 years). There was however no major difference in age between the CRC and adenoma patients. AF is found in 40 (5.4%) in the control group, 73 (7.2%) in adenoma patients and 53 (10.1%) in CRC patients (P for trend = 0.002). Hypertension is found in 368 (50%) in controls, in 528 (52%) in adenoma patients and in 310 (59%) in CRC patients (P = 0.01). The frequency of heart failure was 95 (13%) in controls, 134 (13%) in adenoma and 78 (14%) in CRC patients (P = 0.62). Diabetes was diagnosed in 216 (29%) controls, in 318 (31%) adenoma patients and in 178 (33%) in the CRC group (P = 0.28). The frequencies of alcohol consumption and tobacco use were higher in the control group. In all groups, there was a spike in AF occurrence around 50 years of age with much higher frequencies after 70 years of age (Fig 1). Table 2 indicates that in each group (control, adenoma and CRC) patients with AF were older and had more heart failure diagnosis. Hypertension diagnosis was more frequent in AF in the CRC patients group.

**Table 1:** Distribution of demographic and clinical variables of CRC and adenoma Patients.

	Control	Adenoma	CRC	P Value
	n = 731	n = 1008	n = 527	
Age (years), median(IQR)	56 (48–69)	66 (56–77)	67 (58–78)	<0.001
Male gender (%)	375(51)	502(50)	246(47)	0.26
AF (%)	40(5.4)	73(7.2)	53(10.1)	0.01
Diabetes (%)	216(29)	318(31)	178(33)	0.28
HTN (%)	368(50)	528(52)	310(59)	0.01
Heart Failure (%)	95(13)	134(13)	78(14)	0.62
Alcohol (%)	125(17)	111(11)	63(12)	0.01
Tobacco (%)	225(31%)	251(25%)	147(27%)	0.03
Number of hospital admissions, median(IQR)	1(1–2)	1(1–2)	1(1–1)	<0.001

**Table 2:** Distribution of demographic and clinical variables based on AF and diagnosis group.

	Controls			Adenoma			CRC		
	AF-	AF+	P value	AF-	AF+	P value	AF-	AF+	P value
	n = 691	n = 40		n = 935	n = 73		n = 474	n = 53	
Age, median (IQR)	55 (48–68)	68 (53–78)	<0.001	65 (56–76)	75 (64–82)	<0.001	66 (57–78)	75 (64–82)	<0.001
Male gender (%)	354 (51%)	21 (53%)	0.9	468 (50%)	34 (47%)	0.6	218 (46%)	28 (53%)	0.3
Diabetes (%)	205 (30%)	11 (28%)	0.8	935 (32%)	18 (25%)	0.2	154 (32%)	24 (45%)	0.062
HTN (%)	344 (50%)	24 (60%)	0.2	488 (52%)	40 (55%)	0.7	270 (57%)	40 (75%)	0.009
Heart Failure (%)	79 (11%)	16 (40%)	<0.001	103 (11%)	31 (42%)	<0.001	51 (11%)	27 (51%)	<0.001
Alcohol (%)	120 (17%)	5 (13%)	0.4	102 (11%)	9 (12%)	0.7	56 (12%)	7 (13%)	0.8
Tobacco (%)	220 (32%)	5 (13%)	0.010	234 (25%)	17 (23%)	0.7	133 (28%)	14 (26%)	0.8
Number of hospital admissions, median(IQR)	1 (1–2)	1 (1–2)	0.7	1 (1–2)	1 (1–1)	0.007	1 (1–1)	1 (1–2)	0.3

We then analyzed the association of AF with demographic and clinical variables in each group separately (Table 3). Older age and heart failure were associated with higher risk of AF in controls, adenoma and CRC patients. We combined three groups to assess the adjusted effect of CRC and adenoma on AF (Table 4). In unadjusted model and compared to the control group, the CRC group (OR = 1.9, P = 0.003) but not the adenoma group (OR = 1.3, P = 0.1) was associated with increased risk of AF (Table 4a). After adjusting for effect of significant predictors of AF (from Table 3) including age and heart failure, the risk of AF was not different in adenoma (OR = 1.1, P = 0.7) nor CRC (OR = 1.4, P = 0.1) compared to the control group (Table 4b).

**Table 3:** The association of AF with demographic and clinical variables in each diagnosis group by logistic regression model.

	Controls^1^		Adenoma^2^		CRC^3^	
	OR (95%CI)	P value	OR (95%CI)	P value	OR (95%CI)	P value
Age, (year)	1.03 (1.01–1.06)	0.006	1.04 (1.02–1.06)	<0.001	1.03 (1.01–1.06)	0.008
Heart Failure	5.2 (2.6–10.5)	<0.001	5.3 (3.2–8.9)	<0.001	7.0 (3.7–13.1)	<0.001
Tobacco	0.3 (0.1–0.8)	0.022	NS		NS	

^1^ Age, hypertension, heart failure and tobacco were included in model.

^2^ Age, diabetes, heart failure and number of hospital admission were included in model.

^3^ Age, diabetes, heart failure and hypertension were included in model.

NS: Non-significant

**Table 4:** The association of AF with adenoma and CRC using a logistic regression model.

a) Unadjusted		OR (95%CI)	P value
Diagnosis	Control	1.0	
	Adenoma	1.3 (0.9–2.0)	0.1
	CRC	1.9 (1.3–3.0)	0.003
Age (year)		1.04 (1.03–1.06)	<0.001
Heart Failure		6.4 (4.6–9.0)	<0.001
b) Adjusted*		OR (95%CI)	P value
Diagnosis	Control	1.0	
	Adenoma	1.1 (0.7–1.6)	0.7
	CRC	1.4 (0.9–2.2)	0.1
Age (year)		1.04 (1.03–1.05)	<0.001
Heart Failure		5.5 (3.9–7.8)	<0.001

* From a logistic regression analysis in which diagnosis, age, heart failure and tobacco were entered into the model and tobacco was removed with a P = 0.8

## Discussion

In this study, we compared AF prevalence in hospital admitted patients with CRC or adenoma to colon lesions-free controls. We aimed to define the association between colon neoplasia and atrial fibrillation in African Americans. The key findings from our study are: 1) AF is highly prevalent among CRC patients; 1 in 10 patients had AF in our study, and 2) the predictors of AF in CRC were similar to that in adenoma and other patients after adjustment for AF risk factors suggesting that the increased AF risk in CRC is explained by higher prevalence of AF risk factors.

In our study, AF distribution in all three groups has been reported to be higher in older age. Prevalence of AF doubles with each advancing decade of age, from 0.5% at age 50–59 years to almost 9% at age 80–89 years [23]. Age is well known to be a risk factor for impaired cardiac function and incidence of stroke [24], on the other hand AF is shown to decrease the survival of CRC.

A meta-analysis in different races shows that prevalence of AF in African American is 4.9% which is very similar to our finding of 5.4% in controls [11]and is much lower than 10% risk of AF in CRC patient in current study. Furthermore in unadjusted analysis of AF association with colonic lesions, we found a higher OR of AF in CRC patients when compared to adenoma patients. [21, 22]

In each of three study groups, age and heart failure seem to be the independent risk factors for AF in three groups. This is in addition to tobacco use in the control group. These risk factors are similar to what have been observed in the general population. The higher prevalence of AF in CRC could be explained by differences in age and prevalence of heart failure. In multivariate analysis, there was no relationship between CRC or colorectal adenoma and AF. Although this is contrary to previous studies, it must be noted that our patients’ population was vastly different than those described in previous reports [21, 22, 25–28]. The risk of AF is lower in African American compared to Caucasian [11]. Furthermore, risk of AF is lower in CRC with distant metastasis [22] and CRC is diagnosed at more advance stages in African American [29]. Association between CRC and AF is partly attributed to the effect of surgery [22], African American are less likely to receive standard therapy including surgery for their CRC [30] and CRC patients in current study suffer from range of comorbidities which could contraindicate the surgery.

The main strength of our study was that this was a large cohort of African American patients, a population that has been under-represented in previous similar studies. These patients are at a very high risk for morbidity, secondary to stroke [31, 32] and thus early detection of atrial fibrillation is paramount. Increasing age and history of hypertension have been shown to increase the risk of developing atrial fibrillation [33]. These risk factors are similarly associated with an increased incidence of CRC however, in our study this did not seem to impact on the incidence of AF. Inflammation has been postulated to contribute to an increased risk of both atrial fibrillation and CRC [34–36]. Therefore, it is possible that higher histological grades of CRC will influence the incidence of AF. Unfortunately, accurate records of the histological staging of the patients in our population were not available to us. Colonic lesions correlation with AF revealed that CRC is likely more associated with AF than colon adenomas, although this relationship could be explained by shared risk factors, it could justify to the need of a higher cardiovascular health surveillance of patients with CRC.

There are some limitations in this study, which we attempted to minimize. The study may be under-powered to identify a small association between CRC and AF. Accurate and complete documentation of the patients’ diagnoses were based on physicians’ documentation from 2000 to 2012. It is possible that relevant patients’ data have been missed in the medical records. However, the control group was taken from the same period of time that would have been subject to the same biases. This was a retrospective study and thus some patients’ characteristics were unobtainable. Although we obtained the key characteristics for the purposes of our study, we were unable to adjust the effect of unmeasured confounders. We were unable to determine which pathology occurred first: AF or CRC, thus, our interpretation could not be one of etiology but rather one of association. The histological stage of CRC was unknown and could play an important role in terms of the degree of inflammation and hence risk for atrial fibrillation. Lastly, we did not have data on the exact treatments received by the CRC patients. We are aware that some chemotherapeutic drugs such as 5-Fluorouracil have been associated with an increased risk of supraventricular tachycardia [37–40].

## Conclusions

Our study showed that frequency of AF in CRC patients is double than in general population. Both AF and CRC share common pathways. CRC patients especially older patients, patients with heart failure will benefit from AF screening and close monitoring.

## Supporting Information

S1 Dataset Database for public usage. (XLSX)
